# Biotic interactions contribute more than environmental factors and geographic distance to biogeographic patterns of soil prokaryotic and fungal communities

**DOI:** 10.3389/fmicb.2023.1134440

**Published:** 2023-03-09

**Authors:** Yu Liu, Chengxiang Ding, Xingfu Li, Derong Su, Jing He

**Affiliations:** ^1^College of Grassland, Beijing Forestry University, Beijing, China; ^2^Academy of Animal Husbandry and Veterinary Science, Qinghai University, Xining, Qinghai, China; ^3^Industry Development and Planning Institute, National Forestry and Grassland Administration, Beijing, China

**Keywords:** arid ecosystem, biogeographic patterns, biotic interaction, soil microorganisms, community assembly

## Abstract

Recent studies have shown distinct soil microbial assembly patterns across taxonomic types, habitat types and regions, but little is known about which factors play a dominant role in soil microbial communities. To bridge this gap, we compared the differences in microbial diversity and community composition across two taxonomic types (prokaryotes and fungi), two habitat types (Artemisia and Poaceae) and three geographic regions in the arid ecosystem of northwest China. To determine the main driving factors shaping the prokaryotic and fungal community assembly, we carried out diverse analyses including null model, partial mantel test and variance partitioning analysis etc. The findings suggested that the processes of community assembly were more diverse among taxonomic categories in comparison to habitats or geographical regions. The predominant driving factor of soil microbial community assembly in arid ecosystem was biotic interactions between microorganisms, followed by environmental filtering and dispersal limitation. Network vertex, positive cohesion and negative cohesion showed the most significant correlations with prokaryotic and fungal diversity and community dissimilarity. Salinity was the major environmental variable structuring the prokaryotic community. Although prokaryotic and fungal communities were jointly regulated by the three factors, the effects of biotic interactions and environmental variables (both are deterministic processes) on the community structure of prokaryotes were stronger than that of fungi. The null model revealed that prokaryotic community assembly was more deterministic, whereas fungal community assembly was structured by stochastic processes. Taken together, these findings unravel the predominant drivers governing microbial community assembly across taxonomic types, habitat types and geographic regions and highlight the impacts of biotic interactions on disentangling soil microbial assembly mechanisms.

## 1. Introduction

Arid and semiarid ecosystems occupy more than one third of the Earth’s terrestrial surface (Nielsen and Ball, 2015). The soil microbes present in arid ecosystems play a significant part in numerous ecological functions such as nutrient cycling and biomass production (Pointing and Belnap, 2012). Additionally, given highly susceptible of microbes to fluctuations in the environment, variations in the soil microbial community can serve as indicators of changes in the arid ecosystem (Neilson et al., 2012). Thus, disentangling the biogeographic patterns of soil microorganisms and the factors that shape their communities can enhance our predictive capacity for understanding the dynamics and functions of arid ecosystems.

The study of microbial biogeography aims to uncover the forces that determine the distribution of microorganisms across space and time (Martiny et al., 2006). Deterministic and stochastic processes are two fundamental and separate ecological mechanisms that shape the biogeographic patterns of microbial communities (Nemergut et al., 2013; Stegen et al., 2015). The traditional viewpoint of the “everything is everywhere, but the environment selects” theory suggests that species are selected by the environment, known as environmental filtering (De Wit and Bouvier, 2006). In contrast, the neutral theory holds that there is no difference in suitability or niche among species, and that dispersal processes or ecological drift are crucial in determining microbial community structure at various spatial and temporal scales (Chave, 2004; Venkataraman et al., 2015). Although both niche and neutral processes contribute to the formation of microbial biogeographic patterns (Yuan et al., 2019), debates persist regarding the relative influence of deterministic and stochastic processes in shaping microbial communities within a specific ecosystem. For instance, Jiao et al. (2021) proposed that fungal communities in arid ecosystems are mainly influenced by stochastic processes, while Guo et al. (2020) found that microbial communities are mainly governed by deterministic processes with strong connections to nutrients.

Prokaryotes and eukaryotes show notable distinctions in their morphological traits, growth rates, environmental adjustability and life strategies (Hannula et al., 2017). An intriguing finding is that the microbial community assembly mechanisms differ from prokaryotes to fungi subjected to the same environmental perturbation. For example, a study on the continental scale reported the distinct biogeographic patterns across microbial taxonomic types in forest soils in which bacterial community variations were regulated by dispersal limitation, while the fungal communities were mainly influenced by environmental filtering (Ma et al., 2017). However, Wang et al. (2022) reported that both soil bacterial and fungal community assembly were governed by deterministic processes during environmental disturbance. Given the significance of prokaryotes and fungi on soil ecosystems and possible distinct community assemblies, studies focusing on a certain community provide insufficient evidence for ecologists to characterize microbial biogeographic patterns. In addition, the relative contributions of deterministic and stochastic processes on microbial biogeographic patterns do not differ in taxonomic types, but are also habitat-dependent (Jiao et al., 2022). Therefore, distinguishing differences in community assembly of different taxonomic types across habitats is vital to disentangle the relative importance of deterministic and stochastic processes in regulating microbial biogeography (Sutherland et al., 2013; Zhou and Ning, 2017) and is still subject to ongoing debate (Antwis et al., 2017).

Deterministic processes of microbial community assembly are consisted by environmental filtering and biotic interactions etc. (Chase and Myers, 2011; Isabwe et al., 2018; García‐Girón et al., 2020). Environmental filtering has been proved to have numerous effects on mircobial community assembly in a set of studies while the contributions of biotic interactions still remains rare understand. Biotic interactions, such as competition and mutualisms, could lead to niche partitioning of community members under environmental heterogeneity (Bruno et al., 2003; Kraft et al., 2008). For example, the limited availability of nutrients and the negative impact of one species on another has been proposed as a factor that restricts the coexistence of various species (Becker et al., 2012; Li et al., 2020), which affects the biogeographic patterns of microorganisms (Zhou and Ning, 2017). Metabolic cross-feeding among microorganisms could induce species coexistence that leads to aggregations of microbes (Zelezniak et al., 2015). The priority effect can give an advantage to the first organisms to settle, allowing them to adapt and control resources, making it difficult for later organisms to establish themselves or survive (Gillespie, 2004; De Meester et al., 2016). These studies suggest that microbial interspecific interactions affect community assembly *via* diverse mechanisms. In addition, it is believed that the significant amount of unexplained variation in the change of microbial communities can be attributed to the diverse range of microbial co-occurrence networks and diverse network topological features (Shi et al., 2016; Fierer, 2017). Despite their importance in community assembly, biological interspecific interactions receive less understanding relative to environmental selection.

Previous studies investigating microbial biogeography typically sampled plots in a single region, which may limit the assessment of the impact of climate and terrain features. In this study, we examined the community assembly mechanisms of prokaryotic and fungal communities in 69 plots among three regions with different climates and elevations within a typical arid region in northwest China. The sample sites were further divided into two habitats according to the dominant plant species: Poaceae and Artemisia. Specifically, we aimed to answer two questions: (i) Does a distinct community assembly mechanism exist across taxonomic types, habitat types and spatial distance? (ii) What are the main (environmental selection, dispersal limitation and biotic interaction) factors driving these changes?

Considering the significantly different traits [e.g., cell size affect the dispersal capacity (Shurin et al., 2009; Farjalla et al., 2012)] and environmental sensitivity between prokaryotes and fungi, we hypothesized that (1) the differences in the relative contributions of deterministic and stochastic processes between taxonomic types would be larger than that between habitat types or spatial distance; (2) Environmental filtering (deterministic processes) have more effects on prokaryotes community assembly, dispersal limitation (stochastic processes) is the dominant factor shaping fungal community assembly, and biotic interactions contribute to both microorganisms.

## 2. Method and materials

### 2.1. Site description and soil sampling

The experimental area is located in northwest China between 95°1′ and 108°18′E latitude and 36°40′ and 40°19′N longitude and is the predominant arid area in China. The mean annual temperature in the region ranges from 1.6 to 7°C, and the mean annual precipitation ranges from 50 to 250 mm (Table 1).

**Table 1:** The environmental variations across three regions and two habitats.

Environmental variables	JQ (21)	ET (24)	HX (24)	Artemisia (37)	Poaceae (32)
MAP-G (mm)	29.02 ± 9.3b	43.79 ± 7.51a	43.52 ± 8.27a	34.95 ± 10.17B	41.19 ± 10.4A
MAP (mm)	170.19 ± 54.77b	251.2 ± 41.79a	267.96 ± 51.25a	208.78 ± 62.31B	243.41 ± 62.76A
MAT-G (°C)	19.24 ± 2.69a	19.69 ± 0.42a	12.13 ± 2.6b	6.35 ± 3.28A	6.31 ± 3.65A
MAT (°C)	8.24 ± 2.35b	8.5 ± 0.38a	2.51 ± 2.27c	17 ± 4.02A	16.74 ± 4.41A
PET-G (mm/month)	1527.99 ± 66.15a	1241.02 ± 102.81c	1498.90 ± 41.77b	1417.76 ± 151.28A	1418.40 ± 151.30A
PET (mm/month)	911.10 ± 39.51a	701.29 ± 74.91c	894.66 ± 27.76b	835.07 ± 110.80A	829.32 ± 109.42A
AI-G	59.10 ± 15.16a	28.22 ± 4.66c	35.85 ± 9.22b	38.80 ± 15.32A	41.97 ± 17.81A
AI	5.99 ± 1.43a	2.78 ± 0.50c	3.47 ± 0.95b	3.87 ± 1.56A	4.15 ± 1.83A
NDVI	0.32 ± 0.22a	0.32 ± 0.12a	0.27 ± 0.12a	0.22 ± 0.08B	0.5 ± 0.1A
Altitude (m)	1,545 ± 475b	1,392 ± 94b	3,052 ± 392a	2036 ± 844A	1974 ± 848A
SBD (g·cm^−3^)	1.48 ± 0.14b	1.55 ± 0.11a	1.47 ± 0.11b	1.51 ± 0.12A	1.49 ± 0.13A
SWC (%)	5.89 ± 2.53b	7.97 ± 3.41a	10.16 ± 4.6a	8.03 ± 3.54A	8.24 ± 4.93A
FWC (%)	44.27 ± 5.14a	41.4 ± 4.06b	44.67 ± 4.22a	43.17 ± 4.47A	43.93 ± 5.06A
KS (cm·min^−1^)	0.08 ± 0.07a	0.11 ± 0.08a	0.09 ± 0.08a	0.11 ± 0.08A	0.07 ± 0.05B
EC (μs·cm^−1^)	0.31 ± 0.24a	0.15 ± 0.07b	0.33 ± 0.23a	0.24 ± 0.18A	0.31 ± 0.26A
Salinity (g·kg^−1^)	0.16 ± 0.12a	0.07 ± 0.04b	0.31 ± 0.33a	0.18 ± 0.25A	0.18 ± 0.18A
Clay (%)	5.18 ± 2.93a	3.35 ± 1.53b	5.05 ± 2.43ab	4.03 ± 2.28B	5.51 ± 2.56A
Silt (%)	23.88 ± 15.29a	16.45 ± 6.97a	24.76 ± 14.04a	18.32 ± 11.16B	28.61 ± 13.79A
Sand (%)	70.94 ± 18.04ab	80.2 ± 8.38a	70.19 ± 16.32b	77.66 ± 13.24A	65.88 ± 16.29B
pH	8.19 ± 0.35b	7.85 ± 0.27c	8.47 ± 0.34a	8.17 ± 0.43A	8.16 ± 0.39A
SOM (g·kg^−1^)	10.87 ± 5.57b	7.41 ± 4.41c	13.26 ± 4.4a	9.97 ± 4.31A	11.63 ± 7.01A
TN (g·kg^−1^)	0.81 ± 0.37b	0.91 ± 0.44b	1.33 ± 0.5a	1.12 ± 0.51A	0.82 ± 0.38B
NH_4_^+^ (mg·kg^−1^)	5.6 ± 3a	5.54 ± 3.81ab	4.58 ± 5.58b	4.72 ± 3.53A	6.3 ± 5.5A
NO_3_^−^ (mg·kg^−1^)	3.81 ± 1.28c	5.13 ± 0.64a	4.61 ± 1.1b	4.6 ± 1.11A	4.44 ± 1.24A
AP (mg·kg^−1^)	9.14 ± 7.27b	11.94 ± 6.01a	15.2 ± 7.34a	12.91 ± 7.27A	10.73 ± 7.03A
AK (mg·kg^−1^)	127.56 ± 66.15b	151.26 ± 41.14b	186.25 ± 81.24a	146.06 ± 62.65A	177.92 ± 76.2A

The numbers in parenthesis are the site numbers of each group; the lowercase letters (a–c) indicate significant differences across the three regions; the capital letters (A–B) indicate significant differences between the two habitats. MAP-G, MAT-G, PET-G, AI-G, average precipitation, air temperature, potential evapotranspiration and arid index from June to September. MAP, MAT, PET, AI annual average precipitation, air temperature, potential evapotranspiration and arid index. NDVI, normalized vegetation index; SBD, soil bulk density; SWC, soil water content; FWC, filed water content; KS, hydraulic conductivity conductivity; EC, electricity conductivity; SOM, soil organic content; TN, soil total nitrogen content; TP, soil total phosphorus content; NH_4_^+^, soil ammonium nitrogen contentcontent; NO3^−^, soil nitrate nitrogen content; AP, soil available phosphorus content; AK, soil available potassium content.

**Figure 1:**
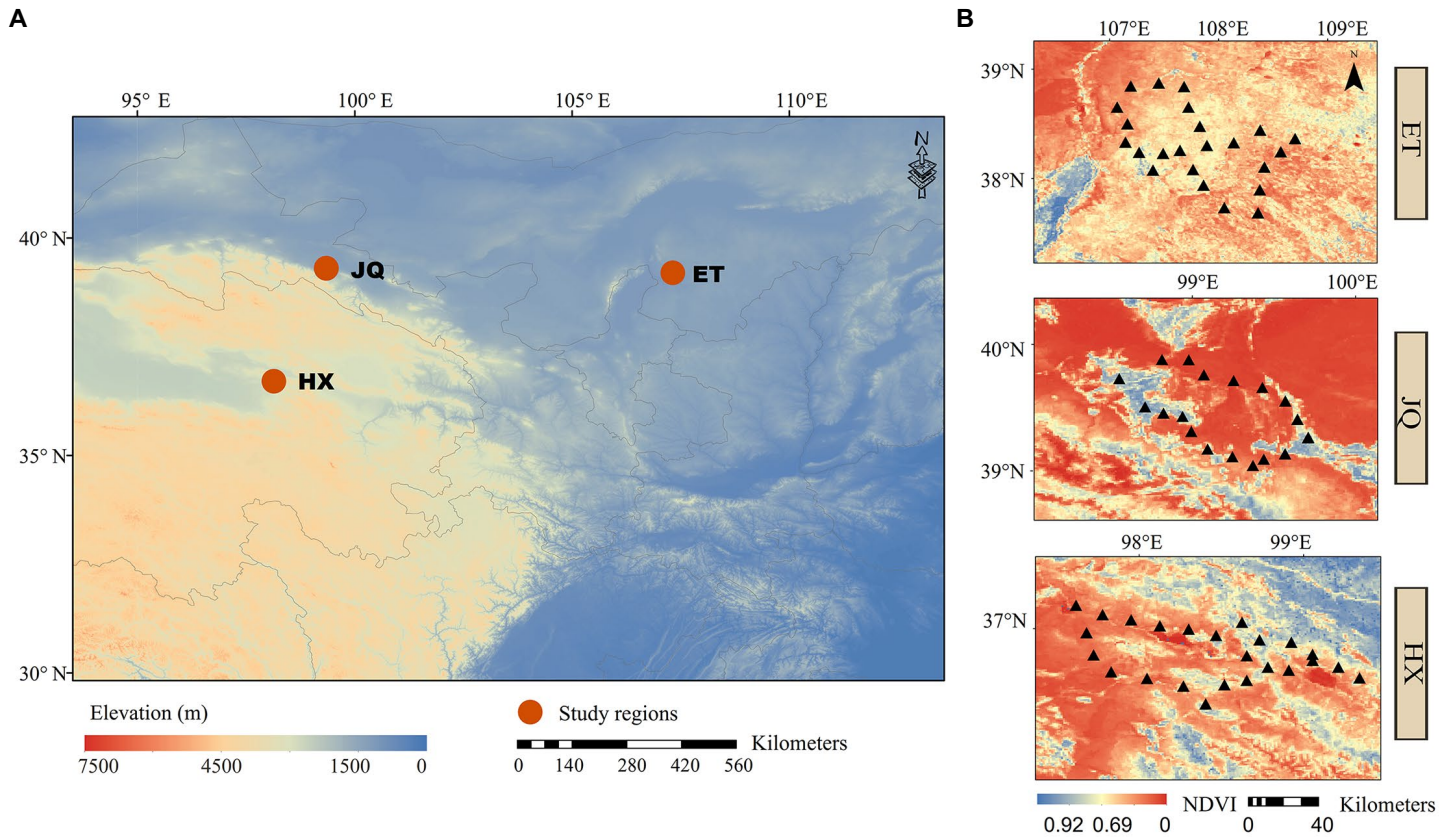
**(A)** The locations of the sample sites. **(B)** The distributions of sampling plots in each regions.

Three arid regions were selected from different provinces, including Haixi in Qinghai Province (HX), Jiuquan in Gansu Province (JQ) and Etoke in Inner Mongolia Province (ET) in northwest China (Figure 1A). The climates are classified as continental plateau climate, continental arid climate and temperate arid climate for HX, JQ and ET, respectively. The ecosystem types are alpine desert grassland, desert grassland and desert grassland for HX, JQ and ET, respectively. The soil types are alpine meadow soil, aeolian sand soil and meadow chestnut soil for HX, JQ and ET, respectively. In each study region (Figure 1B), 24 soil plots were selected (only 21 plots in JQ due to the loss of 3 samples) and all plots were divided into two habitats based on the dominant plant species: Artemisia habitat (37 plots) dominated by *Artemisia ordosica* and *Artemisia annua* and Poaceae habitat (32 plots) dominated by *Stipa* spp., *Leymus* spp., and *Achnatherum* spp. The distance between sites in a region is approximately 20 km. At each plot 5 soil cores (5 cm diametre) from the upper 10 cm were sampled and combined into a single bulk sample, and a total of 69 bulk samples were collected for further analysis. All soil samples were sampled in August 2020 and divided into two subsamples and transported to the laboratory on ice. One subsample was stored at 4°C for measuring soil physicochemical properties, and the other was kept at −80°C for measuring the soil microbial matrix.

### 2.2. Soil physicochemical, plant properties and climate factors

The air-dried soils were used to measure soil total organic matter (SOM) with K_2_Cr_2_O7–H_2_SO4 titrimetric method, soil total nitrogen (TN) and total phosphorus (TP) contents using Kjeldahl digestion and vanadium molybdate yellow colorimetry, respectively. Fresh soils (2.5 g) were extracted with 2 M KCl and filtered to determine soil nitrate (NO_3_^−^) and ammonium (NH_4_^+^) contents using Clever Chem 200+. The particle size composition was determined using a laser particle size analyser (mastersizer2000, Malvern Instruments, United Kingdom). Soil electrical conductivity was measured with a DDS-307 conductivity metre (INESA Scientific Instrument, China). Soil pH was determined using a Sartorius pH meter (PB-10, from Sartorius Corporate Administration GmbH in Göttingen, Germany) by mixing fresh soil with water in a 1:5 ratio. The soil soluble salt content (Salinity) was measured using the residue drying method (Zhang et al., 2019). Soil water content (SWC) was determined as the ratio of the mass of water in the soil sample to the mass of dried soil. Field water capacity (FWC) was calculated as the maximum water content divided by the volume of the soil core. Soil bulk density (SBD) was measured by dividing the mass of oven-dried soil by the volume of the soil core. The saturated hydraulic conductivity (KS) was estimated in the laboratory using a permeameter (Eijkelkamp Agrisearch Equipments, The Netherlands), and the constant head method was used. Data for mean annual air temperature (MAT) and mean annual precipitation (MAP) were obtained from the WorldClim database, while the potential evapotranspiration was obtained from a monthly 1 km dataset (Shouzhang, 2022). The arid index was calculated by dividing the potential evapotranspiration by the mean annual air temperature. The plant index was calculated using Normalized Difference Vegetation Index (NDVI), which was derived from the atmospherically corrected bi-directional surface reflectance data obtained from NASA Land Processes Distributed Active Archive Center’s MOD13Q1 products. The data, which had been masked to exclude water, clouds, heavy aerosols, and cloud shadows, was used to compute NDVI between 9 May and 30 September. The NDVI was calculated using the following equation.


$$NDVI=\frac{\rho_{NIR}-\rho_{RED}}{\rho_{NIR}+\rho_{RED}}$$

where $\rho_{NIR}$ is the reflectance in the near-infrared band (841–876 nm), $\rho_{Red}$ is the reflectance in the red band (620–670 nm).

### 2.3. DNA extraction and sequence processing

Soil DNA was extracted from 0.5 g of frozen soil samples using the FastDNA SPIN Kit for Soil by MP Biomedicals. The quantity of the extracted DNA was determined using a NanoDrop 2000 Spectrophotometer from Bio-Rad Laboratories. Polymerase chain reaction (PCR) was performed using specific primer sets for prokaryotes (515F and 806R) and fungi (ITS1F and ITS2) targeting the ITS1 region. The resulting amplicon was sequenced using the Illumina MiSeq platform and generated approximately 250 bp paired-end reads. The raw sequencing data was analyzed using the QIIME2 software (version 2020.6; Bolyen et al., 2019). The DADA2 plugin in QIIME2 (Callahan et al., 2016) was used to filter low-quality sequences and eliminate chimeras, producing amplicon sequence variants (ASVs). These ASVs were classified using the QIIME2 naive Bayes classifier (Bokulich et al., 2018), which was trained on 99% operational taxonomic units from the SILVA rRNA database (v 132) (Quast et al., 2012) for prokaryotes and the UNITE database for fungi (Nilsson et al., 2019). The raw sequence results were presented in Supplementary Table S1.

### 2.4. Data filtering and alpha and beta diversity analysis

To ensure a more stringent analysis, ASVs with small counts in a limited number of samples were removed given they are likely the results of sequencing errors or low-level contamination. The analysis was based on filtered data, which only included ASVs that occurred in at least 20% of the total samples and had at least 4 counts per sample. The diversity of both the prokaryotic and fungal communities was calculated using the Shannon index for each individual sample. The community dissimilarity, or beta diversity, was estimated using the Bray-Curtis index and was visualized using principal coordinate analysis (PCoA). The statistical differences were calculated using a permutation analysis of variance (PERMANOVA). All of these analyses were performed using R 4.0.1 with the vegan package (Oksanen et al., 2013).

### 2.5. Microbial co-occurrences network analyses

The co-occurrence networks in previous studies were often constructed using subjective thresholds, leading to a lack of objectivity. To address this issue, a random matrix theory (RMT)-based approach was employed to construct prokaryotic and fungal co-occurrence networks objectively using the Molecular Ecological Network Analyses Pipeline^1^ and the constructed networks were visualized using Gephi^2^. The network analysis followed the protocols established in studies by Deng et al. (2012) and Zhou et al. (2010). The network’s topological features were evaluated to determine its complexity. Keystone nodes were identified based on hub nodes including network hubs, connectors, and module hubs, which were classified based on within-module connectivity (Zi) and among-module connectivity (Pi) (Olesen et al., 2007).

Cohesion, a metric based on abundance-weighted pairwise correlations, was calculated using the protocol outlined by Herren and Mcmahon (2017). The metric is calculated as follows:


$$cohesion=\overset{m}{\underset{i=1}{\sum }}abundance_{i}\times conneceness_{i}$$

where m is the total number of taxa in a community and i is the taxa in each sample.

This protocol measures the interconnectedness of a community using a Pearson correlation matrix. The strength of each pairwise correlation was verified using a null model. The average positive and negative null model-corrected correlations were calculated for each sample to obtain a connectedness matrix. Finally, positive and negative cohesions were calculated for each sample using the above formula. Cohesion reflects the degree of cooperative behavior or competition within a community and can serve as a proxy for the strength of biotic interactions if taxa are subject to similar environmental drivers and influenced differently by species interactions. The community cohesions were then used for analyses to determine βNTI and stability measurements.

### 2.6. Community assembly analysis

We adopted a null-model-based approach to assess the role of niche versus neutral processes in shaping microbial communities. This approach has been widely used in previous studies (Stegen et al., 2015; Zhou and Ning, 2017). The Mantel correlogram (using the “mantel.correlog” function in the R package “vegan”) was employed to estimate the correlation between niche differences (calculated as the pairwise Euclidean distances of the environmental optima of ASVs) and phylogenetic distances. The stronger signal observed at short phylogenetic distances (as shown in Supplementary Figure S2) indicated the potential for quantifying phylogenetic turnover using βNTI.

The standardized effect size of this correlation, known as the beta nearest taxon index (βNTI), was calculated as the difference between the observed value and the mean of the null distribution, normalized by its standard deviation (Stegen et al., 2013, 2015).To further distinguish the processes driving community assembly, we calculated the Bray–Curtis based Raup-Crick metric (RCbray). This metric is based on the difference between the observed Bray–Curtis dissimilarity and its null distribution (Dini-Andreote et al., 2015; Stegen et al., 2015). Values of |βNTI| <2 and RCbray > 0.95 indicate dispersal limitation, |βNTI| <2 and RCbray < −0.95 indicate homogenizing dispersal, and |βNTI| <2 and |RCbray| < 0.95 indicate drift. We estimated the relative importance of each process by calculating the fraction of values of βNTI and RCbray within different categories.

### 2.7. Distance-based Moran’s eigenvector maps

Spatial variables are determined by distance-based Moran’s eigenvector maps (dbMEM), which were called principal coordinates of neighbor matrices (PCNM; Borcard and Legendre, 2002), is a standard method for partitioning the effects of the space in ecological studies. First of all, a matrix of dbMEM variables was constructed based on latitude and longitude with the adespatial package (Dray et al., 2017).

The Moran’s I test (Moran, 1950) was utilized to assess the spatial correlation, and only eigenfunctions from dbMEM that showed a positive correlation were included in further analysis (Borcard et al., 2011). Redundancy between Hellinger-transformed prokaryotic and fungal community data (ASVs) and geographic coordinates were used to determine if there are significant linear trend. If there were significant linear trends, detrended analysis was conducted because linear trends masked all other spatial structures during variation partitioning analysis (VPA). The significance of the coefficients of the detrended matrix regression on the dbMEM matrix was tested by conducting 999 permutations of the residuals (Borcard et al., 2011). The selection of significant dbMEM variables was performed through a forward selection process, based on the 999 Monte Carlo permutation procedure of the residuals, using the “packfor” package (Blanchet et al., 2008). The variation partitioning analysis was then conducted using the significant dbMEM variables, in order to determine the relative impact of dispersal limitation on the dissimilarity of microbial communities.

### 2.8. Statistical analysis

The driving factors of microbial community dissimilarity can be divided into three categories, environmental variables, spatial variables (significant dbMEM) and biotic interactions (network topological features). The environmental characteristics included soil properties (i.e., pH and SWC), plant index (i.e., NDVI) and climatic factors (i.e., MAT and MAP). Random forest analysis was conducted to estimate the explained variations in driving factors to prokaryotic and fungal alpha diversity across two biomes based on R^2^ with the randomforest package (Liaw and Wiener, 2002). The significance and impact of each predictor were determined based on the increase in mean square error (MSE) using 999 permutation tests with the rfpermute package (Archer, 2016). Meanwhile, the best regression model based on AIC was used to select the primary factors with the stats package. Then, the line regression model was used to determine their relative influence on microbial Shannon diversity. The distance-decay relationship was measured using ordinary least-squares regressions and Mantel tests between geographic distances and community dissimilarities, and the geographic distances among sites were calculated with the geosphere package (Hijmans et al., 2017). The connection between the variability in the environment and the differences in the microbial communities was calculated using a linear model analysis based on distances with the ecodist package (Goslee and Urban, 2007). The significance of the slope in the relationship between the distance and dissimilarity was evaluated through 999 permutations of residuals. Finally, the relative impact of environmental factors, spatial variables, and biological interactions were determined through a variation partitioning analysis (VPA), which was performed using the vegan package (Oksanen et al., 2013).

## 3. Results

### 3.1. Variations in environmental properties

As shown in Table 1, most of the 26 environmental variables significantly differed across the three regions and two habitats. JQ had the lowest MAP and MAP-G but the highest PET, PET-G, AI and AI-G. HX had the lowest MAT and MAT-G. No significant differences in NDVI were observed among three regions. The altitude of HX was significantly higher than that of the other regions. HX had the highest soil nutrient contents including SWC, SOM, TN, NH_4_^+^, AP and AK, while ET had the highest NO_3_^−^ contents. Soil textures also differed among three regions. ET had the largest percentage of sand (80.2 ± 8.38%) and silt (16.45 ± 6.97%) while the lowest percentage of clay (3.35 ± 1.53%). The highest EC, salinity and pH were observed in HX.

Artemisia and Poaceae had significant differences in MAP and MAP-G while no differences in MAT, MAT-G, PET-G, PET, AI-G and AI. The NDVI was significantly higher in Poaceae (0.5 ± 0.1) than in Artemisia (0.22 ± 0.08). Poaceae had higher NDVI, SWC, EC, Clay, Silt, SOM, NH_4_^+^, and AK contents. Artemisia had higher KS, sand, TN, NO_3_-and AP contents. No significant differences in pH were observed between habitats.

### 3.2. Microbial community composition and diversity across three region and two habitats

The most abundant prokaryotic phyla were Actinobacteria, Proteobacteria, Acidobacteria and Crenarchaeota, which showed distinct distributions among regions but showed no significant differences between biomes (Supplementary Figure S1B). Acidobacteria and Crenarchaeota were more abundant in HX, while the relative abundance of Gemmatimonadetes was significantly higher in ET (Supplementary Figure S1A). Fungal communities also exhibited compositional differences in regions but no differences in habitats. JQ harboured a relatively higher abundance of Ascomycota but had the least abundance of Mortierellomycota, which was highest in ET.

**Figure 2:**
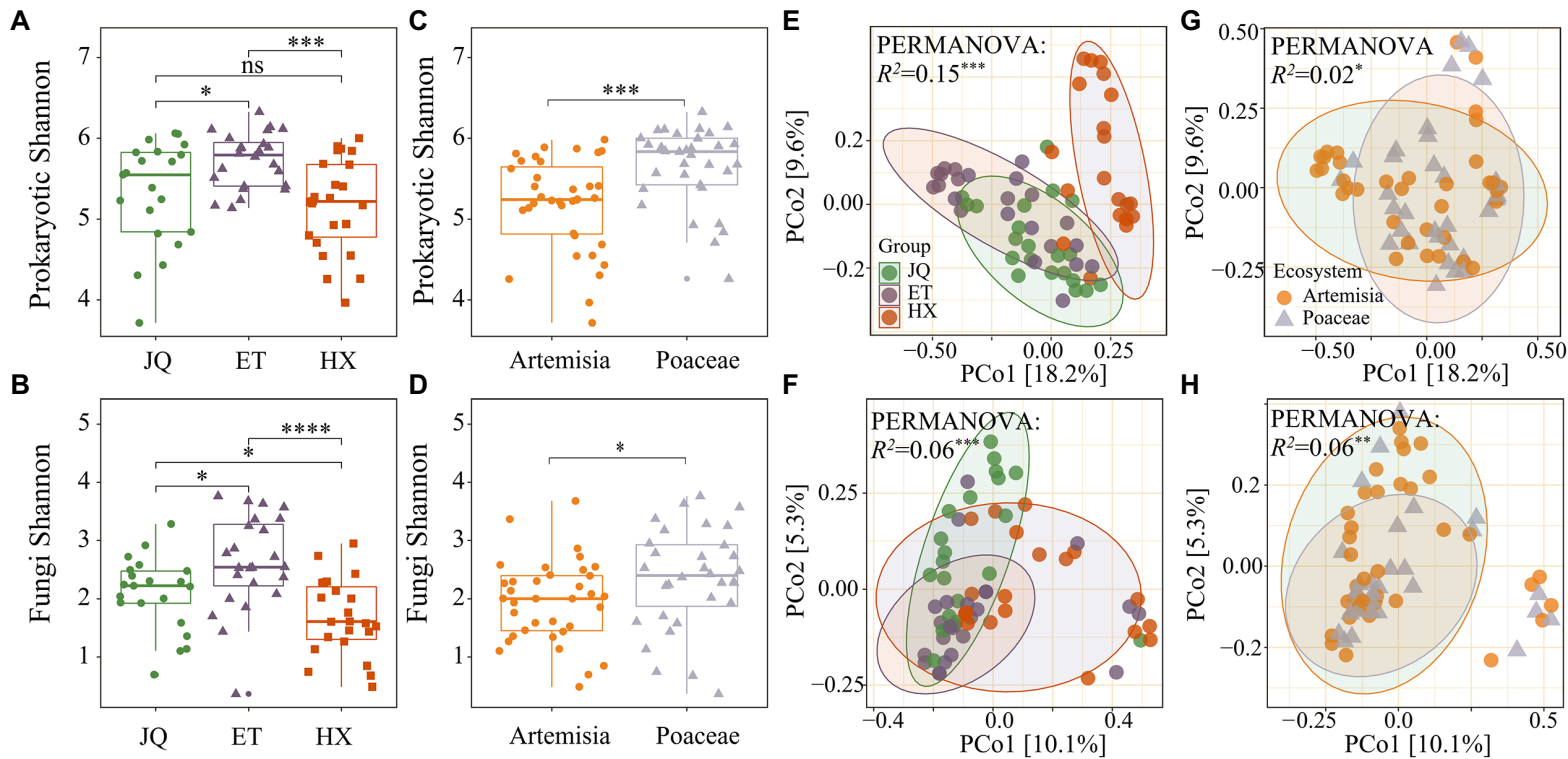
Alpha diversity of prokaryotic and fungal phyla across three regions **(A,B)** and two habitats **(C,D)**. PCoA of prokaryotic and fungal phyla across three regions **(E,F)** and two habitats **(G,H)**. *, **, *** indicate significance at the 0.05, 0.01 and 0.001 levels, respectively.

Shannon’s diversity measurements indicated that prokaryotic communities in the ET were the highest diverse, followed by JQ and HX (Figure 2A). Poaceae had a greater α-diversity than Artemisia (Figure 2C). We found similar patterns in the fungal matrix, and ET had a greater α-diversity in comparison with other regions (Figure 2B). Shannon’s diversity in Poaceae was greater than that in Artemisia (Figure 2D).

PCoA results showed that prokaryotic communities in HX were far away from those in the other samples (Figure 2E), while no distinct separation among the three fungal communities was observed (Figure 2F). We observed no apparent separation of prokaryotic and fungal communities between Poaceae and Artemisia (Figures 2G,H). The results of the PERMANOVA test showed that the composition of both the soil prokaryotic and fungal communities were significantly different across the three regions (*R*^2^ = 0.15, *p* < 0.001 for prokaryotic communities; *R*^2^ = 0.06, *p* < 0.001 for fungal communities) and between two habitats (*R*^2^ = 0.02, *p* < 0.001 for prokaryotic communities; *R*^2^ = 0.06, *p* < 0.001 for fungal communities).

### 3.3. Soil microbial community assembly patterns

**Figure 3:**
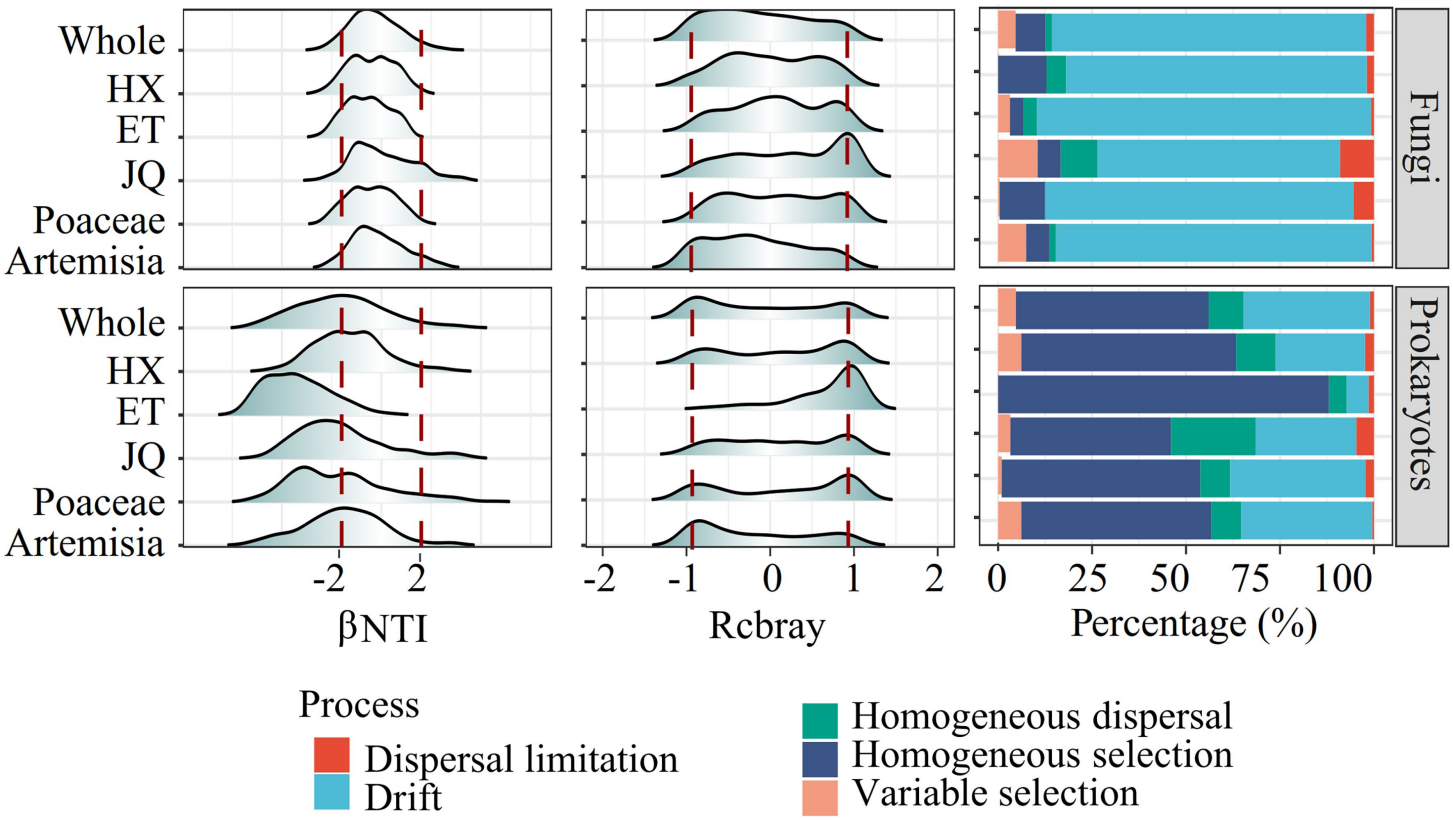
The βNTI, rcbray and the fraction of turnover in the assembly of soil microorganism communities.

The null model revealed distinct assembly patterns between types of microorganisms but similar patterns between regions or habitats. As shown in Figure 3, prokaryotic community assembly in all regions and habitats was mainly regulated by homogeneous selection, with some impact from ecological drift. For fungi, community assembly was largely influenced by ecological drift. This was indicated by the βNTI and Rcbray values, with most of the βNTI values for prokaryotes less than−2 and most for fungi between−2 and 2, and the absolute values of Rcbray less than 0.95 for both prokaryotes and fungi (Figure 3). These findings suggest that deterministic processes were the primary drivers of prokaryotic community assembly, while stochastic processes played a more prominent role in fungal community assembly.

### 3.4. The intra–and cross–kingdom microbial co-occurrence network

**Figure 4:**
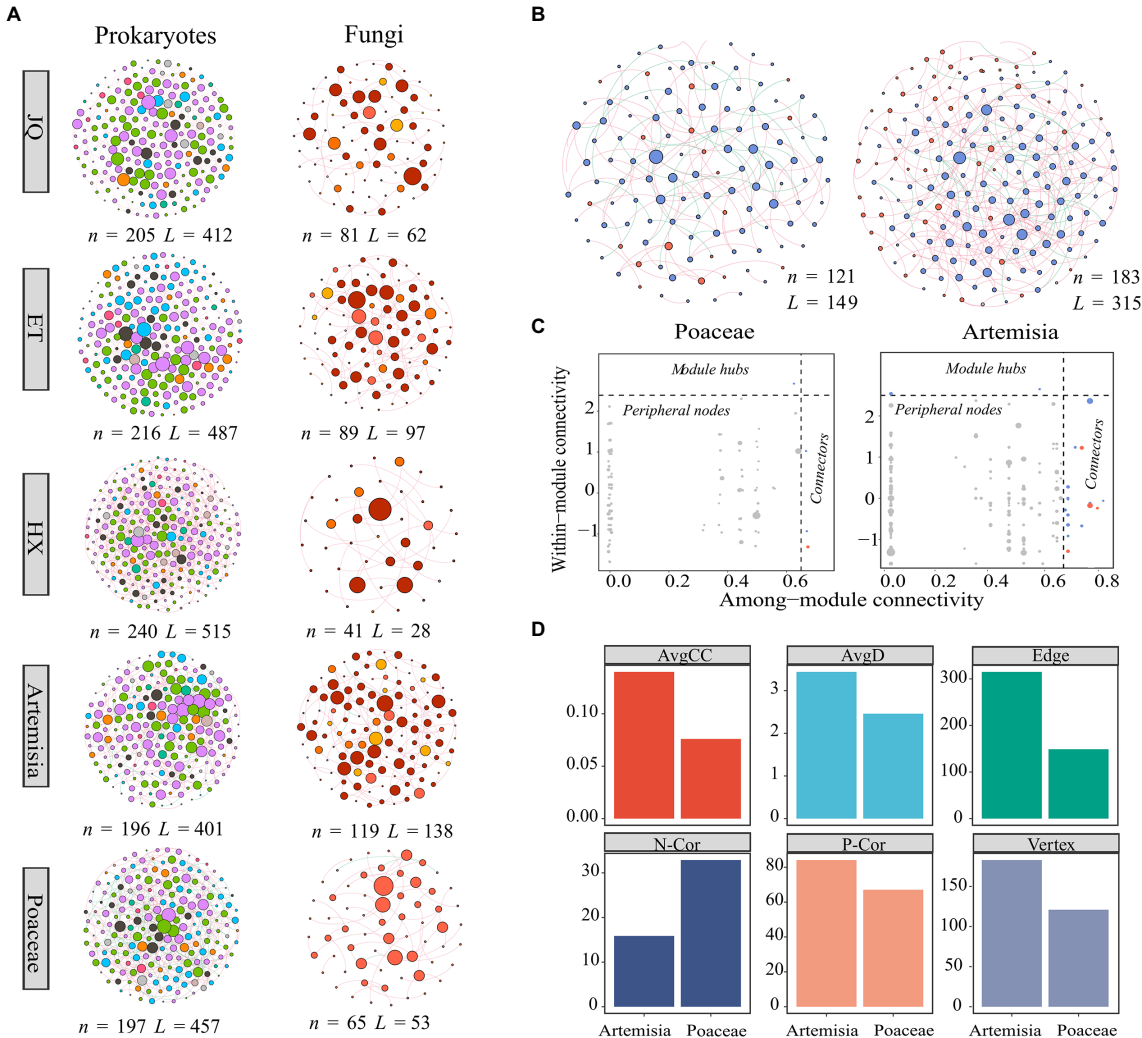
**(A)** Co-occurrence network of prokaryotic and fungal communities across three regions and two habitats. **(B)** Cross-kingdom co-occurrence networks of prokaryotic and fungal communities, blue nodes represent fungal taxa, and red nodes represent prokaryotic taxa. **(C)** Keystone nodes of cross-kingdom network. **(D)** Topological features of the cross-kingdom network. n indicates vertex, L indicates edge, AvgD indicates average degree, AvgCC indicates average cluster coefficient, Heter indicates network heterogeneity, N-Cor indicates the number of negative correlations, and P-Cor indicates the number of positive correlations.

We found distinct differences in network structures between prokaryotic and fungal communities (Figure 4A), whereas relatively few differences were found among regions and between biomes (Supplementary Table S2). Meanwhile, we observed more edges and higher average degrees in prokaryotic networks while more modules in fungal networks, indicating that prokaryotic networks were more connected and fungal networks were more modularized (Supplementary Table S2). We then explored the biotic interactions of soil microorganisms by establishing cross-kingdom co-occurrence networks consisting of prokaryotes and fungal taxa based on correlation matrix. These results showed that fungi-prokaryotes network in Artemisia was more connected and clustered than that in Poaceae.

### 3.5. Primary predictor of soil microbial species diversity

**Figure 5:**
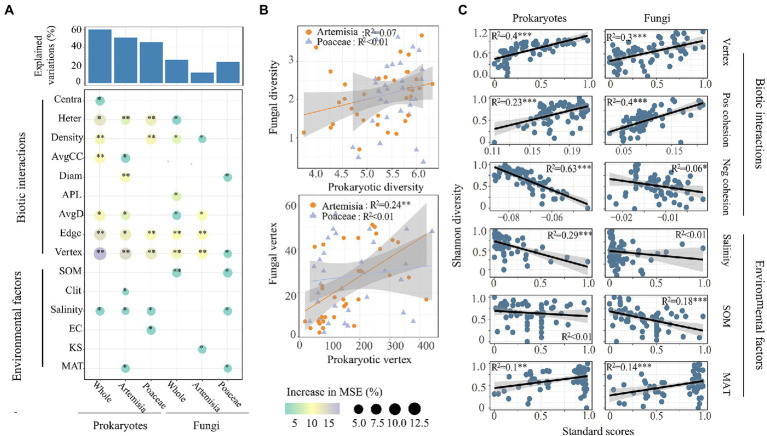
Driving factors of microbial α-diversity across habitats. **(A)** Contributions of different factors to prokaryotic and fungal α-diversity based on random forest model. Circle size and colour represent the variables’ importance. The abbreviations of environmental variables are in accordance with the Table 1. **(B)** Relationships of α-diversity and vertex number between prokaryotic and fungal communities were estimated using linear least-squares regression. **(C)** Relationships between microbial α-diversity and the predominant factors based on the results of the best regression model with the least AIC. Pos Cohesion indicates positive cohesion, Neg Cohesion indicates the negative cohesion.

We found that most network attributes played crucial roles in both prokaryotic and fungal α-diversity, and network vertices had the largest observed effect (Figure 5A). Of the environmental factors, only salinity and MAT affected both prokaryotic and fungal α-diversity. Clay and EC had significant effects on the prokaryotic diversity of Artemisia and Poaceae, respectively. SOM and KS influenced the fungal diversity of Poaceae and Artemisia, respectively (Figure 5A). The species diversity between prokaryotes and fungi had no significant relationship, whereas prokaryotic network vertex increased linearly with fungal network vertex in Artemisia (Figure 5B). Based on the results of the best regression model with the lowest AIC, we found that network attributes including vertex, positive cohesion, negative cohesion were the best predictors for microbial Shannon diversity (Figure 5C).

### 3.6. The relative contributions of environmental variables, geographic distance and biotic interactions To soil microbial community dissimilarity

**Figure 6:**
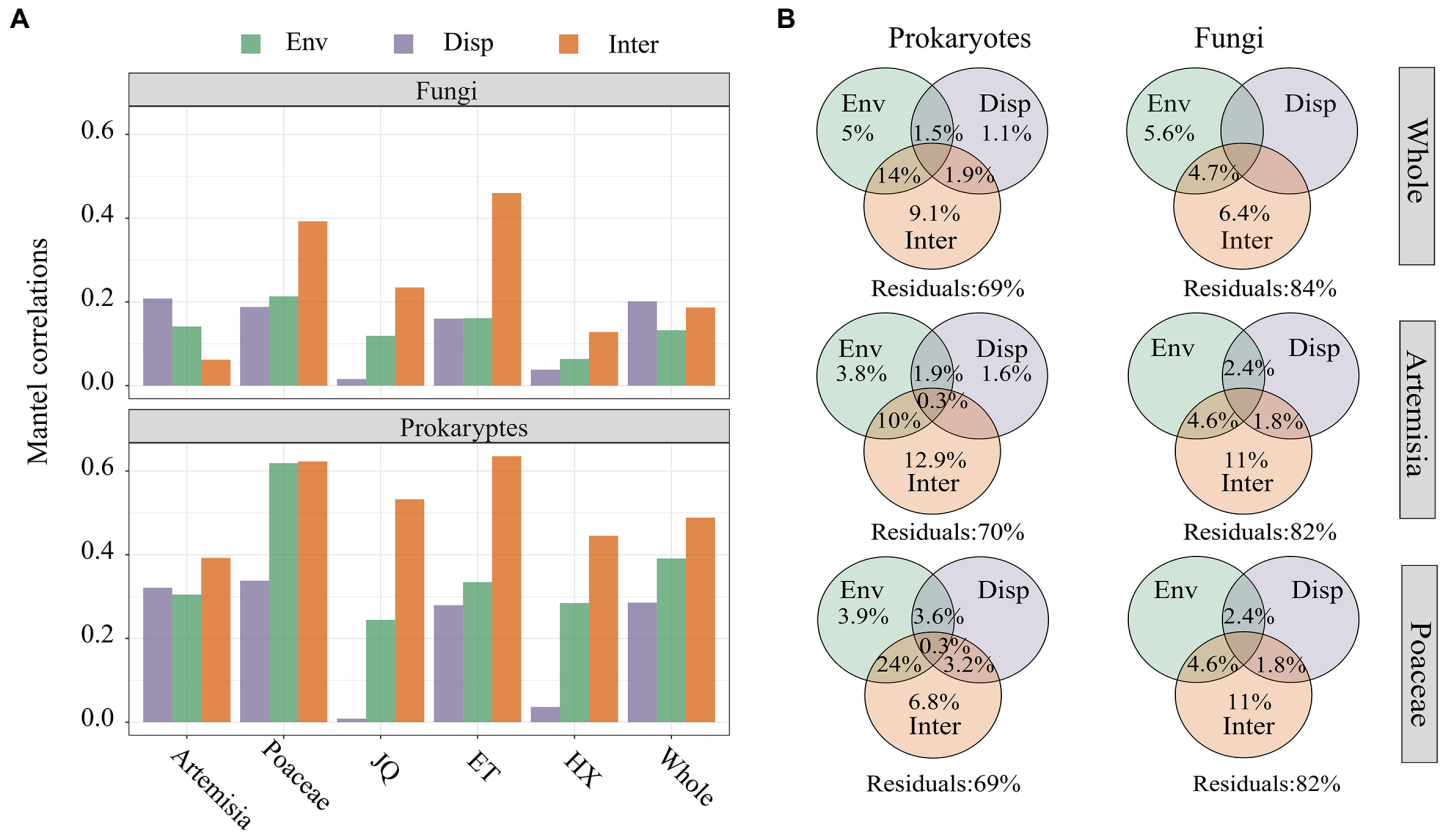
**(A)** Mantel correlations between bray–curtis dissimilarity and βNTI of microorganism and the distance matrix of environmental variables (Env), geographic distance (Disp) and biotic interactions (Inter); **(B)** The variation partition analysis of prokaryotic and fungal communities that can be explained by environmental filtering (Env), dispersal limitation (Disp) and biological interactions (Inter).

The results of Mantel Tests showed biotic interactions (Inter) had higher correlations than environmental matrix (Env) and geographic distance (Disp) with prokaryotic community dissimilarity across all three regions and two habitats (Figure 6A). In the entire dataset and in the Artemisia habitat, the dissimilarity of the fungal community showed stronger associations with geographic distance, whereas the strongest correlations were observed between biotic interactions and fungal community dissimilarity across three regions and in the Proaceae habitat (Figure 6A). Furthermore, the results of the Variance Decomposition Analysis showed that biotic interactions played a greater role in shaping both the prokaryotic and fungal communities than environmental factors and geographic distance, both in the overall dataset, habitats of Poaceae and Artemisia (Figure 6B) and three regions (Supplementary Figure S3).

The results of Partial Mantel Test showed that biotic interactions showed the strongest correlation with both bray–curtis dissimilarity (bray) and phylogenetic turnover (βNTI) of prokaryotic community, followed by environmental factors and geographic distance. Fungal βNTI showed the highest correlation with biotic interactions but fungal bray-curits dissimilarity had higher correlations with geographic distance than biotic interactions (Table 2).

**Table 2:** Partial mantel test results showing comparisons between microbial community dissimilarity, βNTI, and a one-distance matrix while controlling for the other two distance matrices.

Organism	Test	Parameter	Inter (*Controlling for Env and Disp*)	Env (*Controlling for Inter and Disp*)	Disp (*Controlling for Env and Inter*)
Prokaryotes	βNTI	*r*	0.40	0.24	0.038
		*P*	<0.001	<0.001	0.06
	Bray	r	0.34	0.33	0.10
		*P*	<0.001	<0.001	<0.001
Fungi	βNTI	*r*	0.21	0.13	0.08
		*P*	<0.001	<0.001	<0.001
	Bray	r	0.08	0.05	0.14
		*P*	<0.001	<0.001	<0.001

Inter, Env and Disp indicate the distance matrix of network attributes, environmental variables and geographic locations, respectively.

## 4. Discussion

Soil prokaryotes and fungi play primary but distinct roles in ecosystem functioning. Therefore, we separately analyzed them to identify common and differential impacts on species diversity, community turnover, and assembly patterns on a regional scale. Deterministic and stochastic processes are the two most common drivers that shape soil microbial diversity and community structure (the diversity and biogeography of soil bacterial communities). Multiple approaches have been used previously to estimate their relative contributions in soil metacommunities, including variation partitioning analysis (Duan et al., 2022), distance-decay relationships (Hanson et al., 2012) and null-model partitioning (Gotelli, 2000). Given that no certain strategy is inherently superior, we have here adopted diverse methods to determine the relative influence of environmental selection and dispersal limitation on the formation of the microbial community to increase the confidence in the results.

### 4.1. Distinct community assembly patterns of the soil prokaryotes and fungi.

The null model analysis based on βNTI and Rcbray could distinguish the relative contributions of the deterministic process and stochastic process in governing the microbial communities (Stegen et al., 2013, 2015). In this study, most of the βNTI values for prokaryotes were less than−2 and most for fungi were between−2 and 2, suggesting that deterministic processes played a stronger role in prokaryotic community assembly, whereas fungal community assembly was structured by stochastic processes (Figure 3). |Rcbray| < 0.95 further indicated that stochastic processes were dominanted by ecological drift (random proliferation or death of microorganisms). Such differential responses across taxonomic types have also been observed in various habitats, such as soil (Powell et al., 2015), glaciers (Jiang et al., 2018), sediment (Zhao et al., 2022) and freshwater (Logares et al., 2018). Despite significant differences in soil physicochemical properties and spatial distance (Table 1), microorganisms belonging to the same groups of soil microorganisms showed similar assembly modes in different geographic regions or habitats (Figure 3). These results support our first hypothesis that taxonomic type plays a greater role than habitats types and geographic distance on soil microbial community structures.

One possible explanation for the differential response of prokaryotic and fungal communities to geographic distance and habitat types is that they have distinct sensitivities to environmental selection. In addition, increasing environmental heterogeneity can result in greater dissimilarity among microbial communities with increasing geographic scale. The VPA and Partial mantel test showed that changes in habitat environments have a greater impact on community turnover in prokaryotes compared to fungi (Figure 6; Supplementary Table S3), suggesting that prokaryotes are more susceptible to environmental changes than fungi. Moreover, the differences in dispersal capacity of the two groups of microorganisms could also foster the distinct assembly models. Propagule sizes of fungi are typically within the range 5–50 μm diametre (Ingold, 1971) while those of bacteria are usually within 0.2–20 μm diametre (Young, 2006), organisms with smaller propagule size and larger number are typically thought to be easier to spread. Mantel test between distance and community structure revealed the differences of Prokaryotes and fungi in dispersal capacity (Figure 6A). However, large spatial distance could weaken the difference in dispersal capacity in soil between prokaryotes and fungi. A survey reported the limited impact of dispersal capacity in determining the distributions of soil bacteria at global scale (Bahram et al., 2018). The weaker correlations between geographic distances and both prokaryotic and fungal dissimilarities further supported less effects of dispersal limitation on soil microbial community assembly at region or bigger scale (Figure 6A).

Ecological drift, a stochastic process referring to random births and deaths in a population (Vellend, 2010), is expected to be more significant with decreasing population size because random demographic events play a significant role in smaller populations (Fodelianakis et al., 2021). Fungi with a smaller population size relative to prokaryotes hence tend to be affected by the greater role of ecological drift. The results based on null model analysis also suggested that fungal community assembly was dominated by ecological drift. The large part of the unexplained variance of fungal community turnover in VPA may also be due to the unmeasurable stochastic process.

### 4.2. The effects of environmental filtering on microbial community assembly

A recent global-scale study reported that environmental filtering was the predominant driver of soil prokaryotic community turnover (Fierer and Jackson, 2006). We also observed that the community turnover of prokaryotes showed a more significant relationship with the aggregate environmental matrix than that of fungi (Figure 5B). VPA revealed that the environmental matrix explained more variance in community dissimilarity of prokaryotes than that of fungi, further demonstrating the great role of environmental selection in prokaryotic community assembly (Figure 6A). These results further indicate that deterministic processes (environmental filtering) have more impacts on soil prokaryotic community, while soil fungal community were mainly structured by stochastic processes.

The results of the Mantel analysis indicated that salinity was the strongest factor affecting both the βNTI and β-diversity (Supplementary Table S3), which is consistent with results found in desert (Zhang et al., 2019) and lake ecosystems (Yang et al., 2019). The OLS model indicated that higher salinity levels were associated with a reduction in species diversity. This negative relationship possibly due to the heightened extracellular osmolarity resulting from excessive salt concentrations (Oren, 2011; Rath and Rousk, 2015), which could cause the death or inactivity of taxa that cannot tolerate osmotic stress (Pontarp et al., 2012). Furthermore, significant correlations were found between the prokaryotic community and multiple environmental variables (e.g., MAT and AP, Figure 5A; Supplementary Table S3), suggesting that the formation of prokaryotic communities is influenced by various niches. However, it should be noted that a considerable amount of variation in the soil microbial community observed in our study was not accounted for. Possible reasons for this include unmeasured environmental variables, the limited scope of our sampling, and potential biotic interactions (Jiao et al., 2020).

### 4.3. The effects of biotic interactions on microbial alpha and beta diversity

Notably, evaluations of the relative contributions of determinism to community assembly largely concentrate on the set of environmental factors. However, the dominant processes refer not only to environmental selection but also to all ecological forces driving community turnover, such as biotic interactions (Singh et al., 2009). The results of the null model showed that homogeneous selection was dominant in the prokaryotic community assembly, which demonstrated that the biotic interactions existed (Danczak et al., 2018). However, elucidating microbial spatial–temporal distribution from the perspective of interspecific interactions is a tremendous challenge because it is more difficult to directly quantify the interactive patterns of microorganisms relative to macroorganisms. A typical way of including species interactions into an explanation matrix is to apply correlation-based cooccurrence network analysis (Layeghifard et al., 2017).

Random forest analysis revealed that network topological parameters had better prediction performance in both prokaryotic and fungal alpha diversity than environmental variables (Figure 5A). Linear regression analysis found that the number of network vertices and positive cohesion could explain more variance in microbial Shannon diversity (Figure 5C). These findings indicated that network topological features could be an effective proxy for biotic interactions and that biotic interactions play a critical role in driving microbial alpha diversity.

Biotic interactions have been reported a predominant factor in the β-diversity of diazotrophic and bacterial communities in paddy soil (Gao et al., 2019). In this study, the network topological feature matrix had stronger correlations with prokaryotic and fungal β diversity (Figure 6A), which indicates biotic interactions may also be the primary driving in structuring soil prokaryotic and fungal community composition. In addition, we found network attributes had stronger correlations (Figure 6A; Table 2) and explained more variance of both prokaryotic and fungal community structure dissimilarity and phylogenetic turnover than environmental factors and geographic distance (Figure 6B). These results suggested that taxa-taxa interactions may play a more important roles than environmental filtering or dispersal limitation in both soil prokaryotic and fungal community assembly processes.

Community complexity was investigated by relating a recently published measure of cohesion of biotic interactive network to ecological structuring processes (Herren and Mcmahon, 2017). Negative cohesion of microbial co-occurrence network has been reported to be significantly correlated with the β-diversity of bacteria (Herren and Mcmahon, 2017). A study in aquifers found that microbial communities with more negative cohesion values experienced lower turnover and were more likely to regulated by homogenizing selection, whereas less complexity communities experienced higher turnover and susceptibility to stochastic processes (Danczak et al., 2018). In this study, we also found significantly positive correlations between negative cohesion and βNTI values of prokaryotic communities in arid ecosystem (Supplementary Figure S4), indicating not only the connectivity but also the complexity of biotic interactions could affect the microbial community turnover and community assembly processes.

A previous study reported that fungal richness could affect the relative contributions of deterministic and stochastic to bacterial community assembly processes (Jiao et al., 2021). Complex interactions between soil fungi and prokaryotes in the cross-kingdom network were observed in this study (Figure 4). These results may indicate that the assembly mechanism of prokaryotic and fungal communities was influenced not only by intra-kingdom interaction but also by the biotic interactions across taxonomic types (Duan et al., 2022). Despite no significant correlation between the species diversity of fungi and prokaryotes in Artemisia habitat, we found significant positive correlations between their vertex (Figure 5B), which implies the potential facilitation or symbiosis between prokaryotic and fungal taxa (Duan et al., 2022). Therefore, estimation based only on intra-kingdom biotic interactions may introduce bias, and incorporation of cross-kingdom interaction could facilitate the understanding of community assembly, especially in networks with intense inter-kingdom interactions.

It is noteworthy that the effects of biotic interactions were estimated based on the microbial co-occurrence network, which is only a putative species interaction network yielding statistical associations between taxa (Carr et al., 2019). It cannot be too prudent to infer the actual microbial interactions and their ecological meaning. Despite unavoidable defects, the spatiotemporal dynamics of co-occurrence networks hold the potential to affect community assembly and topological features could serve as an effective proxy to estimate the relationships between biotic interactions and ecological processes.

Together, our study revealed the potential of microbial biotic interactions to serve as predictors or interpreters for prokaryotic and fungal α-and β-diversity, built the linkage between diverse microbial co-occurrence network topological features and mechanisms underlying microbial community assembly, and highlighted the importance of biotic interactions including intra-and cross–kingdom interactions, in regulating microbial diversity and microbial community assembly processes across terrestrial ecosystems.

## 5. Conclusion

Our study found that taxonomic differences had a greater impact on community assembly than habitat types or geographic distances. Prokaryotic community assembly was mainly determined by deterministic processes, while fungal community assembly was dominated by stochastic processes. Biotic interactions and environmental filtering were crucial factors in driving deterministic processes for soil prokaryotic communities, with salinity being the main environmental factor affecting prokaryotic diversity and community structure. The strongest correlation with microbial diversity and community structure was observed with network vertex and cohesion, which served as a proxy for biotic interactions. Ecological drift, rather than dispersal limitation, was the main factor driving the stochastic assembly of fungi. This study provides explicit evidence to reveal the major roles of biotic interactions in shaping the assembly processes of microbial communities in region scales. Considering the significant effects of interspecific interactions on microbial community assembly patterns, future empirical and theoretical research are needed to disentangle how taxa-taxa interactions structuring microbial species diversity and community structure facing the increasing global climate changes.

## Data availability statement

The data presented in the study are deposited in the Genome Sequence Archive repository (https://ngdc.cncb.ac.cn/gsa/s/YzfCA6B1), accession number PRJCA014156.

## Author contributions

CD investigated plant ecological diversity and collected soil samples. YL contributed to soil characterization, statistical analysis, data visualization, and wrote the first draft. DS and JH improved the manuscript. All authors contributed to the article and approved the submitted version.

## Funding

This work was supported by the Qinghai Province “Kunlun talents high-end innovation and entrepreneurship talents” project, Independent Research Project of Basic Scientific Research Business Expenses of Qinghai Academy of Animal Husbandry and Veterinary Sciences (mky-2019-03), “Study on the ecological environment monitoring and evaluation system of grassland water-saving in western pastoral areas (2016YFC040030705)” of National key research and development project.

## Conflict of interest

The authors declare that the research was conducted in the absence of any commercial or financial relationships that could be construed as a potential conflict of interest.

## Publisher’s note

All claims expressed in this article are solely those of the authors and do not necessarily represent those of their affiliated organizations, or those of the publisher, the editors and the reviewers. Any product that may be evaluated in this article, or claim that may be made by its manufacturer, is not guaranteed or endorsed by the publisher.

## Footnotes

^1^ http://ieg2.ou.edu/MENA

^2^ https://gephi.org/
