# Supplementary material for: Biotic interactions contribute more than environmental factors and geographic distance to biogeographic patterns of soil prokaryotic and fungal communities
Source: Front Microbiol. 2023 Mar 9;14:1134440. doi: 10.3389/fmicb.2023.1134440 (PMC10034001; doi:10.3389/fmicb.2023.1134440)

Table S1. Sequence information of samples

| ASV Features | Prokaryotes |  | Fungi |
| --- | --- | --- | --- |
| ASV number: | 7644 |  | 6357 |
| ASVs with ≥ 2 counts: | 6608 |  | 1784 |
| Total read counts: | 4407520 |  | 2947323 |
| Average counts per sample: | 63877 |  | 43342 |
| Maximum counts per sample: | 285005 |  | 93323 |
| Minimum counts per sample: | 16192 |  | 10807 |


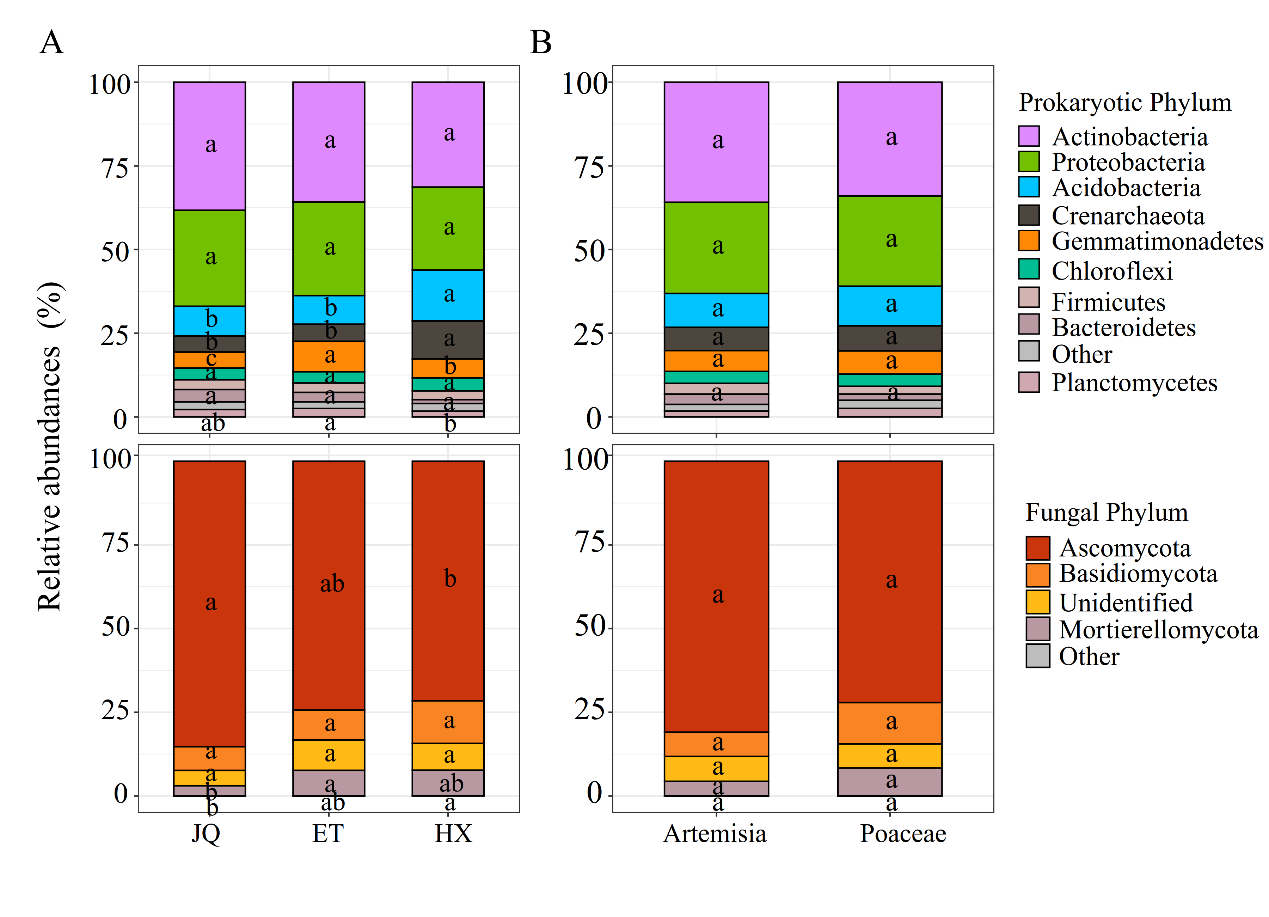


Fig.S1. Relative abundance of the dominant prokaryotic and fungal phyla across three regions (A) and two habitats (B). The lower-case letters indicate the significant differences across regions and habitats.

Table S2. Prokaryotic and fungal network topological properties across three regions and two habitats

| Taxa | Topological properties | Global | JQ | ET | HX | Artemisia | Poaceae |
| --- | --- | --- | --- | --- | --- | --- | --- |
| Prokaryotes | Module | 9.00 | 10.00 | 10.00 | 12.00 | 16.00 | 18.00 |
|  | Vertex | 664.00 | 205.00 | 216.00 | 240.00 | 196.00 | 197.00 |
|  | Edge | 2408.00 | 412.00 | 487.00 | 515.00 | 401.00 | 457.00 |
|  | AvgD | 7.25 | 4.02 | 4.51 | 4.29 | 4.09 | 4.64 |
|  | APL | 5.06 | 4.23 | 4.08 | 4.18 | 0.35 | 0.10 |
|  | Diam | 13.00 | 9.00 | 8.00 | 9.00 | 11.00 | 9.00 |
|  | AvgCC | 0.40 | 0.08 | 0.10 | 0.09 | 0.12 | 0.10 |
|  | Density | 0.01 | 0.02 | 0.02 | 0.02 | 0.02 | 0.02 |
|  | Heter | 1.22 | 0.45 | 0.46 | 0.52 | 0.51 | 0.46 |
|  | Centra | 0.08 | 0.03 | 0.03 | 0.04 | 0.03 | 0.04 |
|  | PosC | 0.18 | 0.16 | 0.20 | 0.17 | 0.18 | 0.17 |
|  | NegC | -0.07 | -0.07 | -0.07 | -0.07 | -0.06 | -0.07 |
| Fungi | Module | 17.00 | 20.00 | 13.00 | 15.00 | 20.00 | 18.00 |
|  | Vertex | 132.00 | 81.00 | 89.00 | 41.00 | 119.00 | 65.00 |
|  | Edge | 793.00 | 62.00 | 97.00 | 28.00 | 138.00 | 53.00 |
|  | AvgD | 12.02 | 1.53 | 2.18 | 1.37 | 2.32 | 1.63 |
|  | APL | 2.89 | 2.48 | 9.09 | 2.11 | 7.53 | 4.88 |
|  | Diam | 7.00 | 8.00 | 22.00 | 6.00 | 18.00 | 13.00 |
|  | AvgCC | 0.64 | 0.05 | 0.12 | 0.15 | 0.15 | 0.10 |
|  | Density | 0.09 | 0.02 | 0.02 | 0.03 | 0.02 | 0.03 |
|  | Heter | 1.14 | 0.49 | 0.57 | 0.51 | 0.50 | 0.56 |
|  | Centra | 0.26 | 0.03 | 0.04 | 0.07 | 0.03 | 0.05 |
|  | PosC | 0.10 | 0.09 | 0.13 | 0.08 | 0.12 | 0.09 |
|  | NegC | -0.01 | -0.01 | -0.01 | -0.01 | -0.01 | -0.01 |

Note: AvgD indicate average degree, APL indicate the average path length, Diam indicate network diameter, AvgCC indicate average cluster coefficient, Heter indicate network heterogeneity, Centra indicate network centralization, PosC indicate the positive cohesion, NegC indicate the negative cohesion.


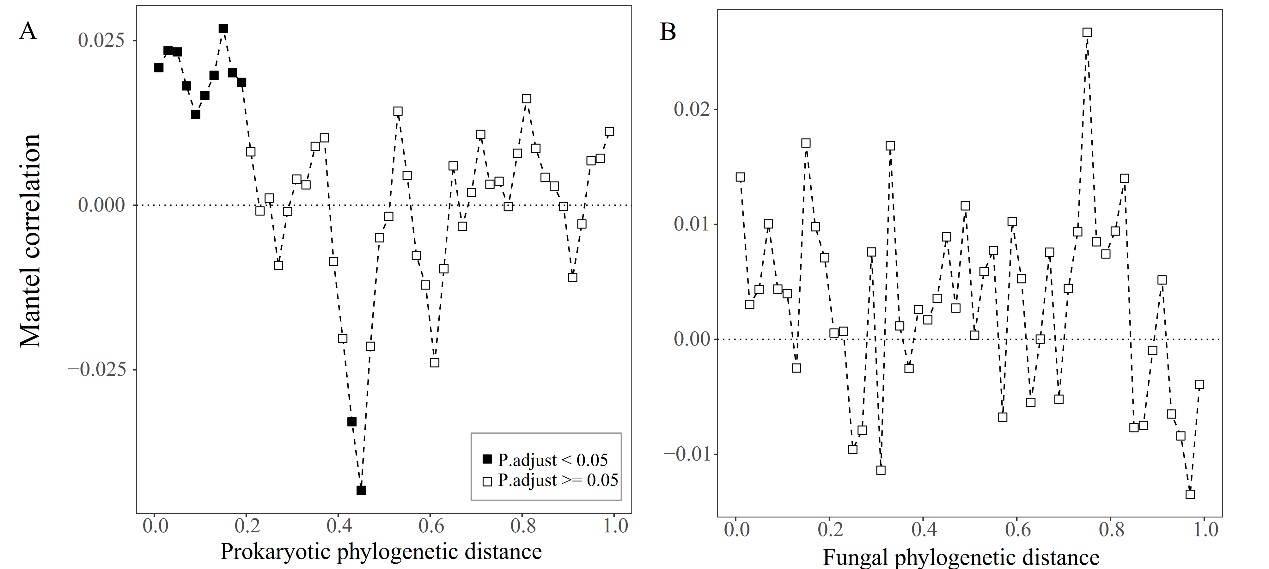


Fig.S2 The mantel correlations between prokaryotic (A) and fungal (B) phylogenetic distance and environmental matrix.

Table S3. Mantel test for the relationships between the β-diversity and βNTI for the soil microbial communities and the environmental variables.

| Environmental variables | Prokaryotic  βNTI | Prokaryotic  β-diversity | Fungal  βNTI | Fungal  β-diversity | |
| --- | --- | --- | --- | --- | --- |
| MAP | -0.06 |  |  |  | |
| MAT | 0.33 | 0.36 |  | 0.18 | |
| NDVI | 0.06 | 0.06 |  | 0.14 | |
| Bulk | 0.07 | 0.06 | 0.05 | 0.06 | |
| SWC | 0.08 | 0.08 | -0.08 | -0.05 | |
| SatWater | 0.07 | 0.06 | 0.05 | 0.06 | |
| Salinity | 0.47 | 0.47 | -0.11 | 0.07 | |
| Clay | 0.17 | 0.19 |  | 0.05 | |
| Silt | 0.2 | 0.2 | -0.07 | 0.06 | |
| Sand | 0.19 | 0.2 | -0.06 | 0.06 | |
| pH | 0.04 | 0.08 | 0.04 | 0.08 | |
| SOM | 0.07 | 0.05 |  | 0.08 | |
| TN | 0.1 | 0.12 | -0.04 |  | |
| NH_4_^+^ | 0.05 | 0.06 |  |  | |
| NO_3_^-^ | 0.05 | 0.11 |  | 0.11 | |
| AP | 0.19 | 0.15 | -0.1 |  | |
| AK | 0.16 | 0.13 | -0.09 |  |  |

Note: the blanks are the insignificant (*P* > 0.05) correlations

Fig.S3 The variation partition analysis of prokaryotic and fungal communities across three regions that can be explained by environmental filtering (Env), dispersal limitation (Disp) and biological interactions (Inter).


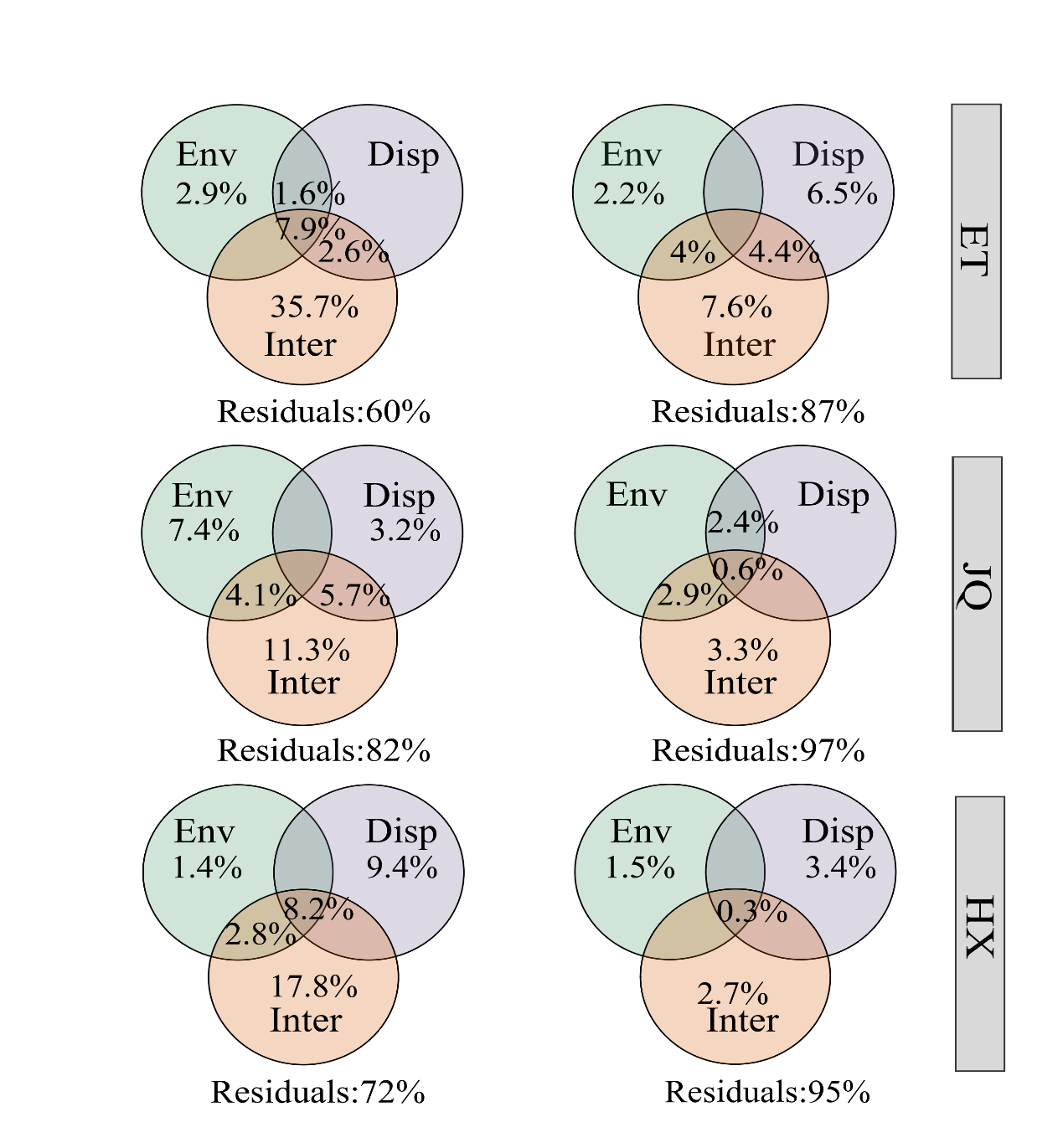


Fig. S4 The relationships between network cohesion and βNTI


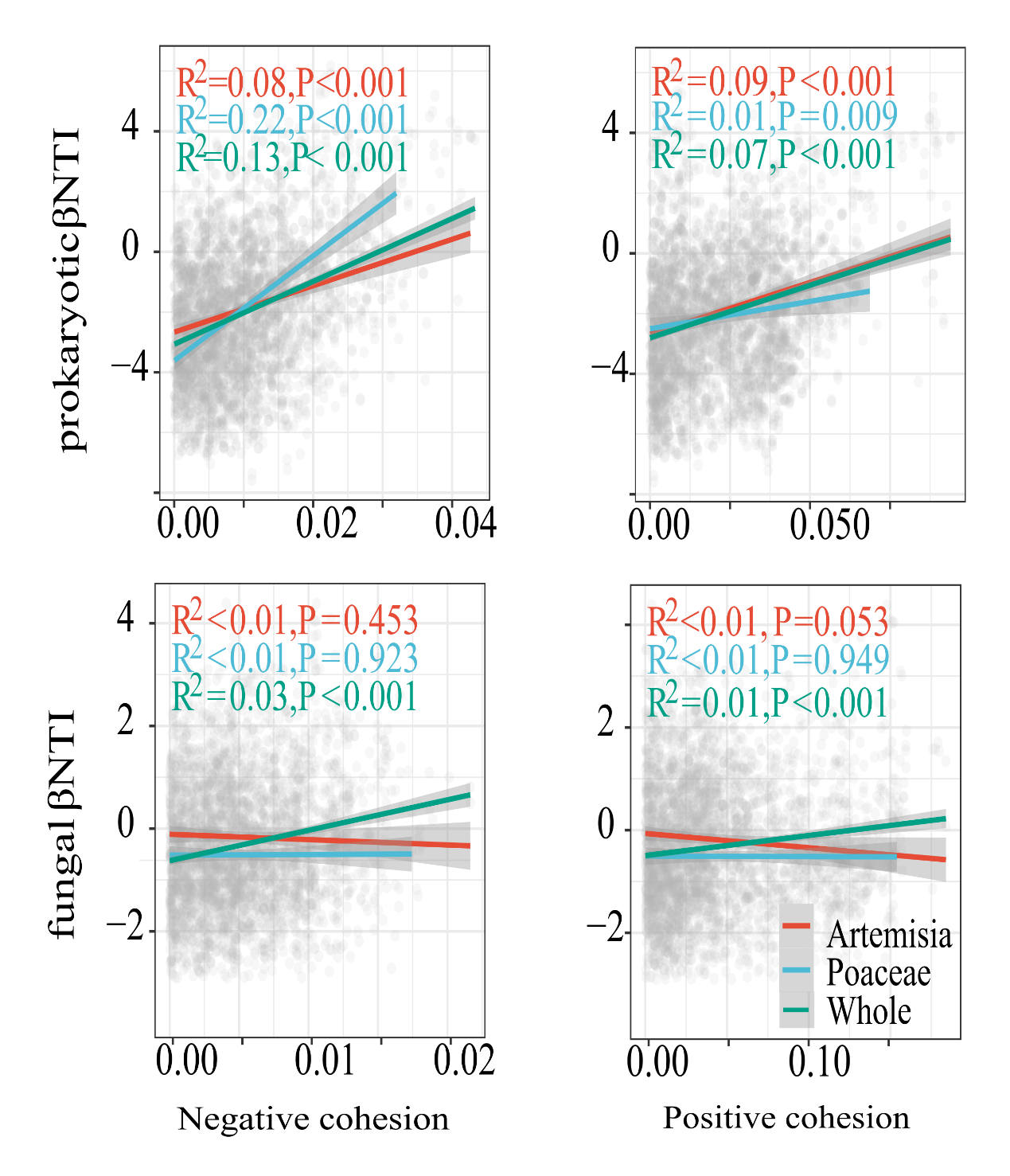

Supplement: Supplementary file 1 [file Data_Sheet_1.docx]
